# Targeted Therapies Modulating Mesenchymal–Epithelial Transition-Linked Oncogenic Signaling in the Tumor Microenvironment: Comparative Profiling of Capmatinib, Bemcentinib, and Galunisertib

**DOI:** 10.3390/jcm14196853

**Published:** 2025-09-27

**Authors:** Piotr Kawczak, Igor Jarosław Feszak, Tomasz Bączek

**Affiliations:** 1 Department of Pharmaceutical Chemistry, Faculty of Pharmacy, Medical University of Gdańsk, 80-416 Gdańsk, Poland; tomasz.baczek@gumed.edu.pl; 2 Institute of Health Sciences, Pomeranian University in Słupsk, 76-200 Słupsk, Poland; igorfeszak@gmail.com; 3 Department of Nursing and Medical Rescue, Institute of Health Sciences, Pomeranian University in Słupsk, 76-200 Słupsk, Poland

**Keywords:** MET, EMT, EMP, targeted treatment, METi, AXLi, TGF-βi

## Abstract

The mesenchymal–epithelial transition/plasticity (MET/EMP) axis is a key regulator of tumor development, cancer progression, and resistance to therapy, making it an attractive target for intervention. This review highlights strategies to modulate MET/EMP using three representative agents—capmatinib, bemcentinib, and galunisertib—each acting on distinct signaling pathways. Capmatinib is a selective MET tyrosine kinase inhibitor with notable efficacy in non-small cell lung cancer harboring MET exon 14 skipping mutations. Bemcentinib blocks AXL receptor tyrosine kinase, interfering with AXL/GAS6 signaling that promotes tumor survival, metastasis, and therapeutic resistance. Galunisertib inhibits TGF-β signaling, reducing epithelial–mesenchymal transition (EMT), immune evasion, and metastatic potential. We discuss their mechanisms of action, therapeutic applications, and current clinical progress. Although these targeted therapies show potential to overcome resistance and improve patient outcomes, challenges remain due to the complex regulation of EMP. Future directions focus on refining combination strategies and advancing personalized approaches to enhance efficacy across multiple cancer types.

## 1. Introduction

Cancer continues to be one of the primary causes of morbidity and mortality worldwide, with metastasis responsible for nearly 90% of cancer-related deaths [1]. Metastatic progression arises from the dissemination of tumor cells from primary tumors to distant organs, a process driven by dynamic and reversible alterations in cellular phenotype and behavior [2]. At the core of this adaptability is epithelial–mesenchymal plasticity (EMP), which encompasses a continuum between epithelial and mesenchymal states, including both epithelial–mesenchymal transition (EMT) and mesenchymal–epithelial transition (MET) [3,4,5].

EMP is clinically critical, as it enables tumor cells to lose epithelial polarity and adhesion [6,7], acquire motility and invasiveness [8,9], and penetrate surrounding tissues [10], thereby facilitating dissemination, intravasation, survival in circulation, immune evasion, and therapy resistance. Conversely, MET—the reacquisition of epithelial features—is frequently required for metastatic colonization and outgrowth at distant sites [11,12,13]. Importantly, MET is an actively regulated process with direct consequences for disease progression and patient outcomes [14]. Beyond metastasis, EMP equips tumor cells to adapt to microenvironmental stressors, evade immune detection, and resist therapy [15]. Although the molecular mechanisms underlying EMT are well characterized, MET remains comparatively underexplored [16,17], despite its clear therapeutic importance.

Several signaling pathways, including TGF-β [18,19], HGF/MET, and AXL [20,21,22], are central regulators of EMP [23,24,25]. Because these pathways govern both EMT and MET, targeting them represents a promising strategy to modulate EMP and potentially improve clinical outcomes. Three targeted inhibitors have emerged as leading EMP modulators: capmatinib (a MET inhibitor), bemcentinib (an AXL inhibitor), and galunisertib (a TGF-β receptor I kinase inhibitor). Bemcentinib blocks AXL, a major EMT driver linked to immune evasion and resistance, thereby promoting MET and enhancing responsiveness to immune checkpoint blockade [26,27,28,29,30,31,32]. Galunisertib inhibits TGF-β receptor I kinase, counteracting TGF-β–induced EMT, restoring epithelial traits, and sensitizing tumors to chemotherapy and immunotherapy [33,34,35,36,37]. Capmatinib suppresses aberrant MET-driven signaling, often activated by MET exon 14 skipping mutations that promote EMT, metastasis, and resistance, thereby reducing EMT and metastatic progression in non-small-cell lung cancer (NSCLC) and showing early improvements in progression-free survival (PFS) in MET-altered tumors [38,39,40,41,42,43,44].

Despite encouraging progress, significant challenges remain in translating EMP-targeted therapies into clinical practice. The heterogeneity of EMP complicates biomarker development and patient stratification [45,46], while compensatory signaling reduces the efficacy of single-agent approaches [47]. Resistance mechanisms, such as secondary mutations and pathway reactivation, further undermine therapeutic durability [48,49,50]. Additionally, because mesenchymal-like states can confer context-dependent survival benefits, the timing and setting of MET induction must be carefully optimized [14,51,52].

Emerging technologies—including single-cell RNA sequencing [53,54,55], lineage tracing [56,57], and patient-derived organoid models [58,59]—are advancing the mapping of EMP’s spatiotemporal dynamics and revealing additional regulatory mechanisms. Epigenetic modifiers [60], microRNAs [61], long non-coding RNAs [62], and tumor microenvironment (TME) components [63] also influence EMP-driven metastasis and therapeutic response.

Therapeutic induction of MET may offer advantages beyond simply reversing EMT. Whereas EMT promotes dissemination, MET facilitates epithelial re-differentiation, restricts metastatic progression, and enhances therapy sensitivity [37,64]. EMT is now understood as a continuum, generating hybrid epithelial/mesenchymal states [65] that support collective migration, immune evasion [66], and drug resistance [67,68]. Targeting these intermediate states through MET induction may destabilize plasticity, mitigate resistance, and improve clinical outcomes [69].

In this review, we synthesize current understanding of MET as a dynamic and targetable process in metastasis. We highlight capmatinib, bemcentinib, and galunisertib as case studies of EMP-modulating therapies, focusing on their mechanisms, therapeutic potential, and the obstacles that remain for clinical translation.

**Figure 1:**
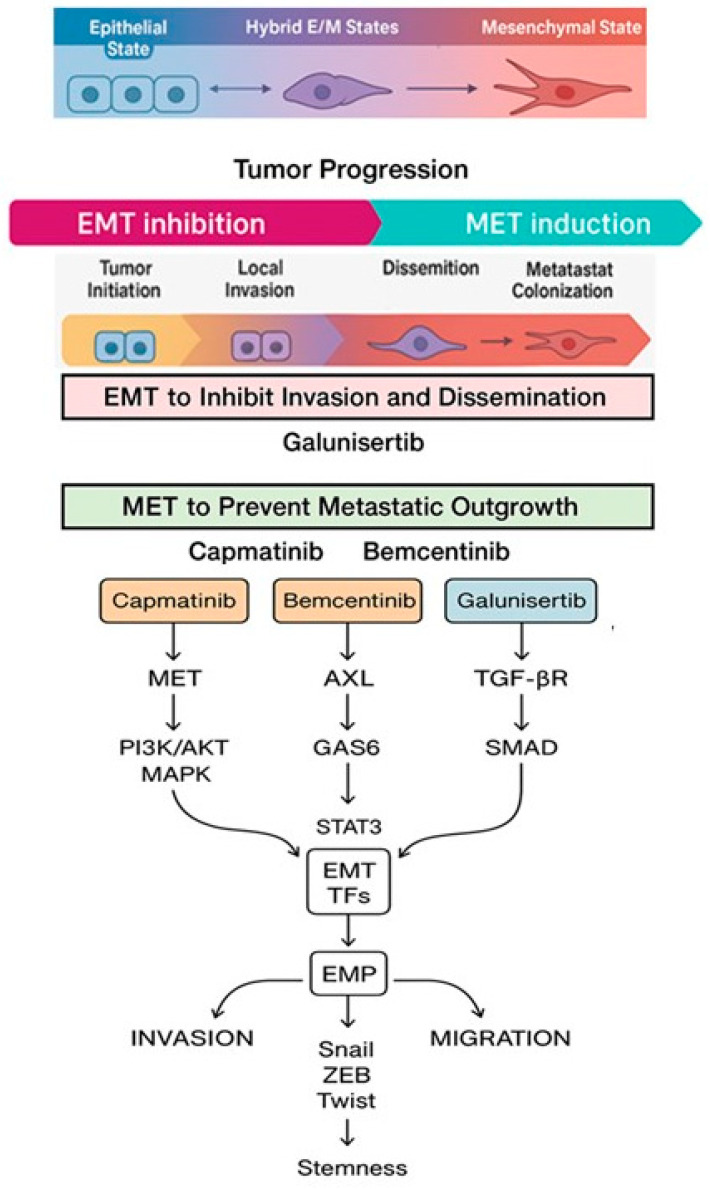
The dynamic epithelial–mesenchymal transition spectrum, highlighting the intermediate hybrid E/M state and its key molecular regulators—including EMT transcription factors (such as SNAIL, SLUG, ZEB1/2), epigenetic modulators, and signaling pathways (such as TGF-β, MET, and AXL)—while a schematic timeline beneath maps tumor progression stages and aligns therapeutic strategies (e.g., early EMT inhibition with galunisertib and later MET targeting with capmatinib or bemcentinib) to emphasize the timing-dependent, reversible nature of EMT in cancer progression. All abbreviations employed are defined in the text in the Abbreviations section.

Figure 1 illustrates the EMT spectrum, hybrid states—molecular regulators and therapeutic timing for EMT/MET modulation throughout tumor progression taking into account similarities and differences of capmatinib, bemcentinib and galunisertib, while Table 1 summarizes the involvement of epithelial–mesenchymal transition transcription factors (EMT-TFs) in the regulation of tumour cell motility (TCM).

## 2. Capmatinib–MET Inhibitor

Capmatinib (INC280) is an orally bioavailable, selective type Ib inhibitor of the MET receptor tyrosine kinase, a critical oncogenic driver involved in cellular proliferation, survival, migration, and invasion [71].

**Figure 2:**
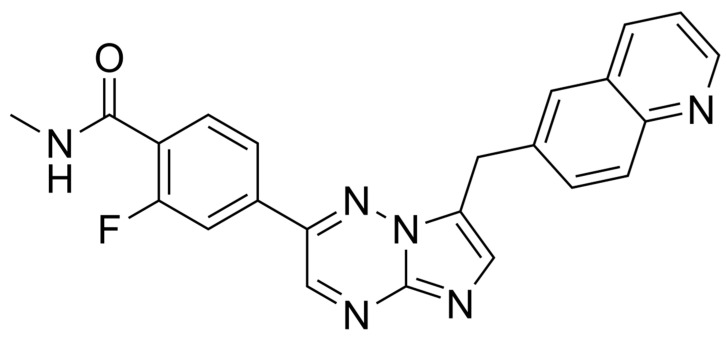
Structural formula of capmatinib.

Figure 2 illustrates the chemical structure of capmatinib.

The MET proto-oncogene encodes a receptor activated by hepatocyte growth factor (HGF), triggering PI3K/AKT, RAS/MAPK, and STAT signaling cascades [72]. Aberrant MET activation promotes tumorigenesis through multiple mechanisms, including exon 14 skipping mutations [73,74], gene amplification [75,76], protein overexpression [77,78], and gene fusions [79,80], particularly in NSCLC. MET exon 14 skipping mutations impair receptor degradation, sustaining oncogenic signaling, and are most frequently detected in NSCLC [81,82,83,84,85], though also observed in gastric [86], renal [87], head and neck [88], and hepatocellular carcinomas (HCCs) [89,90,91,92,93]. Beyond NSCLC, expression of the L1-MET retrotransposon has been linked to bladder and liver cancers [73], colorectal cancer [74], and aggressive breast cancers [75]. Capmatinib binds the ATP-binding pocket of MET, blocking autophosphorylation and downstream activation [24,94,95]. It is administered orally at 400 mg twice daily, metabolized primarily via CYP3A4 and aldehyde oxidase, with a terminal half-life of ~6 h and steady-state levels reached within 15 days [96,97,98]. Importantly, capmatinib penetrates the blood–brain barrier, enabling activity against CNS metastases in NSCLC [99,100].

Preclinical studies demonstrated that capmatinib effectively inhibits MET-driven signaling, reduces EMT, and curtails metastatic spread. Synergistic activity has been shown when combined with inhibitors of compensatory RTKs such as EGFR and AXL [101], as well as with epigenetic modulators like HDAC and DNMT inhibitors that reverse EMT-associated transcriptional programs [102,103].

Clinically, the most notable evidence for capmatinib comes from the phase II GEOMETRY mono-1 trial. In this study, treatment-naïve NSCLC patients harboring MET exon 14 skipping mutations achieved a 68% objective response rate (ORR), while previously treated patients achieved a 41% ORR, with median response durations of 12.6 and 9.7 months, respectively [104,105]. These results supported the accelerated FDA approval of capmatinib (Tabrecta^®^) in May 2020 for metastatic NSCLC with MET exon 14 skipping mutations [106,107,108]. Ongoing studies are evaluating capmatinib in combination with other targeted therapies, such as EGFR inhibitors, and with immunotherapy, to overcome resistance and expand clinical indications [109,110,111]. Early-phase trials are also exploring its role in HCC, glioblastoma, prostate cancer, and papillary renal cell carcinoma, all of which harbor MET pathway aberrations [112,113,114,115,116,117].

Clinically, capmatinib is now established as a first-line targeted therapy for metastatic NSCLC with MET exon 14 skipping mutations, where it produces significantly improved survival compared to chemotherapy [118,119]. Its ability to penetrate the CNS is particularly important given the prevalence of brain metastases in advanced NSCLC [100,109]. At present, evidence largely derives from single-arm trials rather than randomized controlled trials, but outcomes consistently demonstrate durable responses and manageable safety. Expanding applications in other MET-driven cancers remain under active investigation [112,113,114,115,116,117].

Resistance to capmatinib arises through multiple mechanisms. Secondary MET kinase domain mutations, particularly D1228 and Y1230, reduce inhibitor binding and account for ~5–15% of resistance events in MET-driven NSCLC [120,121,122,123]. Additional mechanisms include bypass signaling via EGFR, AXL, HER3, and FGFR1 [124,125,126,127,128], as well as EMT-driven plasticity and epigenetic reprogramming that sustain mesenchymal phenotypes [129,130,131]. Therapeutic strategies to overcome resistance include combining capmatinib with AXL inhibitors such as bemcentinib (NCT04811176) or EGFR inhibitors such as osimertinib (NCT05468697), both of which have shown promising preliminary results with manageable toxicity and encouraging disease control rates [99,132]. Epigenetic approaches, including HDAC and DNMT inhibition, have also been shown to resensitize resistant cells by reversing EMT-associated transcriptional programs [102,103].

Capmatinib’s development highlights the potential role of biomarker-guided therapy in advancing precision oncology, though its broader impact will require further clinical validation [133,134]. Ongoing efforts focus on expanding its indications [135], refining patient selection [136], addressing acquired resistance [137], and implementing multidisciplinary care models to optimize outcomes [138,139,140].

**Figure 3:**
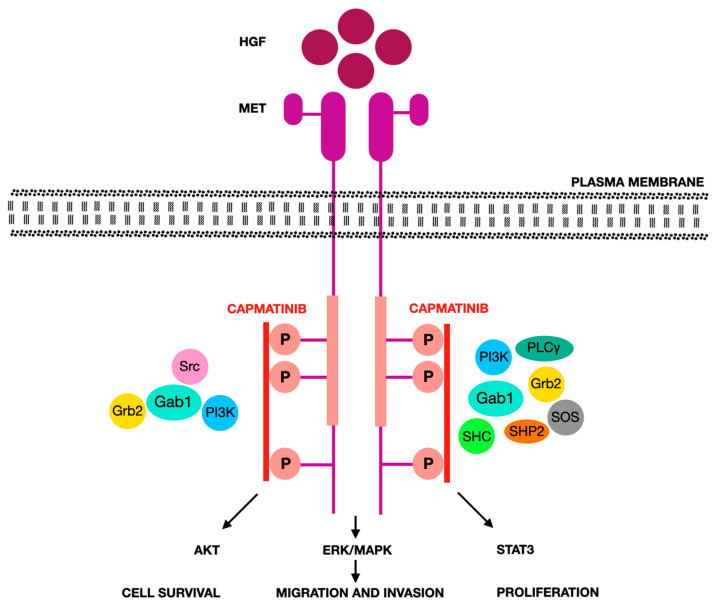
HGF/MET pathway activation according to [141]. When hepatocyte growth factor (HGF) binds to the MET receptor, it triggers structural alterations that promote receptor dimerization and mutual phosphorylation of tyrosine residues within MET’s kinase domain, as well as phosphorylation of tyrosines in the C-terminal region. These phosphorylated sites serve as anchoring points for various adaptor proteins and kinase effectors. Activation of MET subsequently initiates downstream signaling cascades, including the MAPK, PI3K/AKT, and STAT3 pathways, which are involved in regulating cell growth, survival, movement, and invasive behavior in a MET-dependent manner. The MAPK pathway is a signaling cascade that regulates cell growth and survival. It is triggered by receptor activation and proceeds through sequential activation of RAS, RAF, MEK, and ERK. The PI3K/AKT pathway controls cell survival and metabolism. Activation of PI3K leads to the formation of PIP3, which activates AKT, promoting anti-apoptotic and growth signals. The STAT3 pathway regulates gene expression. STAT3 is phosphorylated, dimerizes, and translocates to the nucleus to activate genes involved in proliferation and survival. All abbreviations employed are defined in the text in the Abbreviations section.

Figure 3 depicts the HGF/MET signaling cascade and capmatinib’s mechanism of inhibition, while Table 2 outlines selected cytoplasmic MET inhibitors and their mechanisms.

## 3. Bemcentinib–AXL Inhibitor

Bemcentinib (BGB324, R428) is a highly selective, orally bioavailable small-molecule inhibitor targeting AXL, a receptor tyrosine kinase of the TAM family (TYRO3, AXL, MER) [143,144,145].

**Figure 4:**
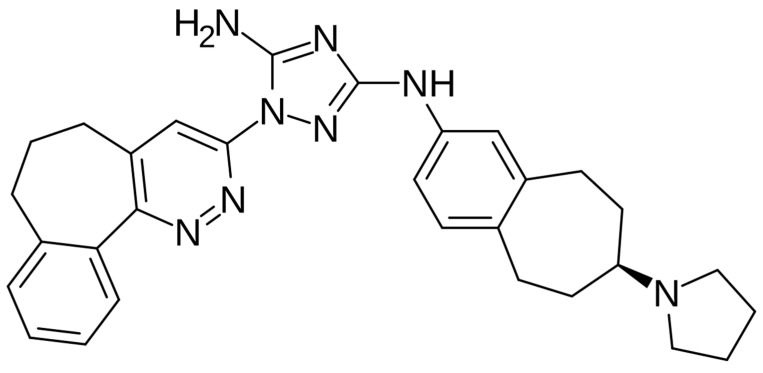
Structural formula of bemcentinib.

Figure 4 illustrates the chemical structure of bemcentinib.

AXL regulates proliferation, survival, migration, immune modulation, and lymphangiogenesis [146,147]. Its activation by the ligand GAS6 induces receptor dimerization and autophosphorylation, initiating PI3K/AKT, MAPK/ERK, and NF-κB signaling pathways that drive tumor progression, metastasis, and immune evasion [50,148]. Unlike TGF-β–driven EMT, which primarily involves SMAD-dependent transcriptional repression, AXL promotes EMT through sustained PI3K/AKT, NF-κB, and STAT3 signaling [149,150,151]. This route fosters phenotypic plasticity, therapy resistance, and stable mesenchymal states that are less reversible [4].

AXL frequently functions as a bypass resistance mechanism in cancers exposed to EGFR, VEGFR, and BRAF inhibitors, where therapeutic pressure induces AXL upregulation and compensatory survival signaling [150,151]. Preclinical studies show that pharmacological inhibition of AXL reverses resistance phenotypes and restores sensitivity to targeted agents [143]. Aberrant AXL signaling is implicated in NSCLC, breast cancer, AML, pancreatic cancer, and other malignancies, with overexpression correlating with poor prognosis and metastatic potential [150,152]. Beyond oncology, AXL activity contributes to fibrotic diseases, motivating trials in chronic kidney disease [153]. Bemcentinib exerts its effects by selectively binding the ATP-binding pocket of AXL, blocking autophosphorylation and downstream signaling [154,155]. This inhibits tumor proliferation, invasion, and metastasis, while promoting EMT reversal [156,157]. It also remodels the TME, enhancing dendritic cell activity, reducing regulatory T cells, and improving responsiveness to immune checkpoint blockade [158,159].

Developed by BerGenBio ASA in the mid-2010s [160,161], bemcentinib demonstrated potent AXL inhibition and antitumor activity in preclinical NSCLC and breast cancer models, both as monotherapy and in combination with other drugs [162,163]. In NSCLC, AXL blockade restored sensitivity to EGFR inhibitors by preventing compensatory survival signaling [164,165]. Early-phase clinical trials established favorable tolerability and preliminary efficacy in AXL-overexpressing malignancies [166,167]. In the phase II ACHILES trial, bemcentinib combined with docetaxel improved PFS and response rates compared to docetaxel alone in treatment-resistant NSCLC [168,169,170,171].

Beyond NSCLC, bemcentinib is in clinical development for AML, pancreatic and gastric cancers, HCC, mantle cell lymphoma, melanoma, endometrial cancer, rhabdomyosarcoma, CNS tumors, and myeloproliferative neoplasms [172,173,174,175,176,177,178,179,180,181,182,183,184,185,186,187,188,189,190]. Administered orally once daily, it has favorable pharmacokinetics characterized by rapid absorption, high bioavailability, and hepatic metabolism via CYP3A4, requiring monitoring for drug–drug interactions [191,192,193,194]. Steady-state plasma concentrations are achieved within days, ensuring sustained AXL inhibition [195,196]. Target engagement is confirmed in biopsies via reduced phosphorylated AXL levels [197,198]. Combination studies with immune checkpoint inhibitors such as pembrolizumab and nivolumab highlight its immunomodulatory potential [199,200].

The safety profile of bemcentinib is manageable. Common toxicities include fatigue, nausea, diarrhea, and mild transaminase elevations [201,202]. Less frequent but notable toxicities include hepatotoxicity and pneumonitis, requiring monitoring [203,204]. Grade 3 ALT/AST elevations have been reported [177,205], while gastrointestinal effects are usually mild [192,206]. Rare events include rash, hypersensitivity, and MRONJ [207,208,209]. Combination use with immune checkpoint inhibitors can lead to immune-related adverse events [210,211], and reproductive safety precautions are warranted [155,212]. Bemcentinib has been granted FDA Fast Track designation for patients with STK11-mutant metastatic NSCLC, a subgroup with poor response to other therapies [213,214,215,216].

Despite its promise, acquired resistance to bemcentinib remains a challenge. Compensatory signaling through parallel pathways or AXL kinase domain mutations can limit efficacy [49,217,218]. Predictive biomarkers are therefore needed, with candidates including AXL expression by IHC, circulating GAS6 levels, mesenchymal gene signatures, and ctDNA [219,220]. Emerging data suggest survival benefits in refractory cancers such as NSCLC and AML [173,221]. Optimizing its use will require biomarker-driven patient selection, multidisciplinary care, and toxicity management [191,222].

Table 3 outlines therapeutic strategies targeting AXL activation to inhibit EMT and overcome drug resistance.

Bemcentinib demonstrates efficacy across AXL-dysregulated cancers, with oral bioavailability and a tolerable safety profile supporting integration into standard therapy [224,225,226]. Ongoing studies aim to overcome resistance, broaden indications, and define its immunomodulatory roles beyond oncology [227]. As a first-in-class AXL inhibitor, bemcentinib exemplifies precision oncology by targeting tumor-intrinsic AXL signaling while modulating the immune microenvironment [228,229]. Its adoption will likely depend on rational combinations and resistance management, with preliminary evidence suggesting it may have activity relevant to both tumor biology and immune evasion, though confirmation in broader clinical settings is still needed [230,231,232,233,234,235,236,237].

**Figure 5:**
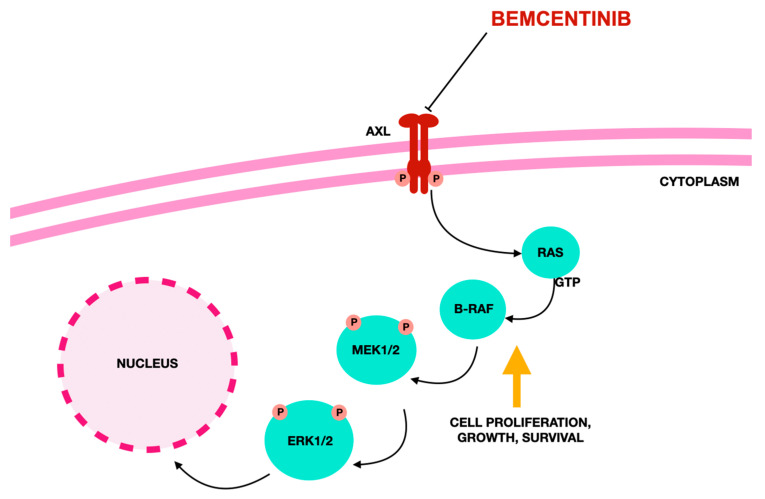
The mechanism of action of bemcenitinib according to [238].The TAM receptor tyrosine kinase family—comprising TYRO-3, AXL, and MER—regulates key cancer-related processes such as cell growth, survival, migration, and metastasis. Among them, AXL is strongly associated with tumor progression, therapy resistance, and immune evasion. The AXL gene, located on chromosome 19q13.2 and made up of 20 exons, encodes a receptor with extracellular IgG-like and fibronectin III domains, a transmembrane region, and an intracellular kinase domain. Increased AXL expression causes resistance to therapies targeting B-RAF (B-Raf proto-oncogene, serine/threonine kinase), a gene that encodes a protein kinase involved in cell growth signaling and is often mutated in melanoma, and also diminishes the effectiveness of PD-1 blockade treatments. All abbreviations employed are defined in the text in the Abbreviations section.

Figure 5 depicts the molecular mechanism of action of bemcentinib.

## 4. Galunisertib–TGF-β Inhibitor

Galunisertib (LY2157299 monohydrate) is a selective, orally bioavailable inhibitor of transforming growth factor-beta receptor type I (TGF-βRI/ALK5), developed by Eli Lilly for oncology applications [239].

**Figure 6:**
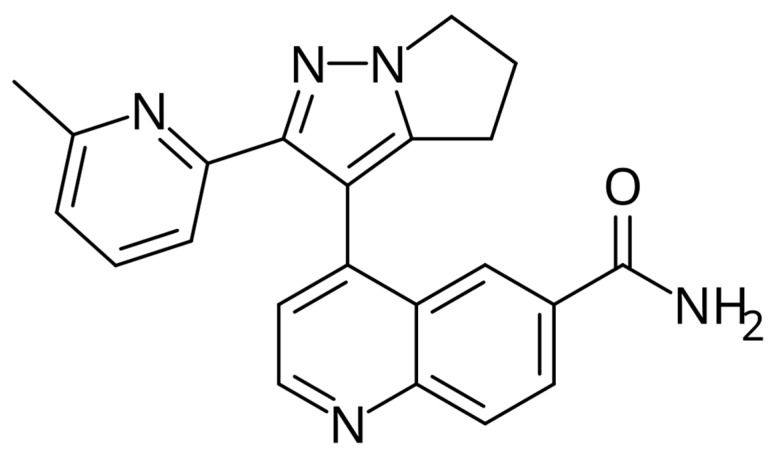
Structural formula of galunisertib.

Figure 6 illustrates the chemical structure of galunisertib.

Its mechanism targets the canonical TGF-β pathway, in which ligand binding to type II receptors triggers phosphorylation of TGF-βRI, activating SMAD2/3 and their association with SMAD4 to form a transcriptionally active complex [240,241,242]. This complex regulates gene expression programs that control proliferation, differentiation, immune responses, and extracellular matrix remodeling [243,244]. In early tumorigenesis, TGF-β signaling exerts tumor-suppressive effects through induction of growth arrest and apoptosis by activating CDK inhibitors such as p15INK4b and p21CIP1 while repressing oncogenes like c-MYC [40,245]. As cancers progress, however, genetic and epigenetic alterations such as TP53 or PTEN loss, oncogenic Ras activation, and microenvironmental factors reprogram TGF-β into a pro-tumorigenic driver of invasion, immune evasion, and EMT via SMAD-dependent and non-canonical PI3K/AKT, MAPK, and Rho GTPase pathways [149,246].

Table 4 provides an overview of the TGFβ signaling pathway.

Galunisertib competitively occupies the ATP-binding pocket of TGF-βRI, blocking kinase activity and preventing SMAD2/3 phosphorylation [248,249,250,251]. By selectively inhibiting this receptor, galunisertib suppresses transcriptional programs that promote tumor progression while sparing many physiological TGF-β functions [252]. Preclinical models across diverse tumor types demonstrated that galunisertib reduces TGF-β signaling, tumor growth, and metastasis [253,254,255,256,257,258,259,260,261]. These findings translated into early-phase trials that confirmed tolerability, characterized pharmacokinetics, and revealed preliminary antitumor activity in biomarker-enriched patient populations [262,263].

In HCC, galunisertib downregulates VEGF, reduces angiogenesis, and suppresses hypoxia-driven pathways by decreasing TGF-β1 activity, hypoxia-inducible factor-1α (HIF-1α), and VEGF protein expression [264,265,266,267,268]. Within TGF-β–enriched (TMEs), galunisertib reduces fibrosis, EMT-related gene expression, and immune evasion, supporting disease stabilization in advanced HCC [269,270,271,272]. In glioblastoma, combining galunisertib with temozolomide and radiotherapy enhanced responses partly by modulating immunosuppressive stroma [273,274,275]. In pancreatic cancer, galunisertib disrupted tumor–stroma interactions, potentiating chemotherapy [276,277,278]. Ongoing trials in NSCLC are exploring galunisertib with immune checkpoint inhibitors and cytotoxics [279,280], while additional studies are underway in colorectal, breast, and melanoma cancers [281,282,283,284].

Beyond oncology, TGF-β signaling regulates fibroblast activation, EMT, and chronic inflammation, linking it to fibrotic diseases such as idiopathic pulmonary fibrosis, diabetic nephropathy, Crohn’s disease, and myocardial fibrosis [285,286,287,288,289,290]. These contexts mirror desmoplastic stroma in pancreatic ductal adenocarcinoma, HCC, and other solid tumors, characterized by excessive extracellular matrix deposition and immune exclusion. Inhibition of TGF-β signaling attenuates fibroblast activation, normalizes ECM, and reduces collagen accumulation, thereby improving perfusion and immune infiltration. Small-molecule inhibitors like galunisertib and vactosertib demonstrate antifibrotic and stroma-modulatory activity in both fibrotic and tumor-bearing models [291,292,293,294,295], providing a strong rationale for combining TGF-β blockade with immune checkpoint inhibitors or cytotoxics to enhance efficacy.

Galunisertib is generally well tolerated, with manageable toxicities including gastrointestinal symptoms (nausea, vomiting, diarrhea, anorexia), fatigue, mild hematologic effects, and transient liver enzyme elevations [296,297,298,299]. Initial concerns about cardiac toxicity, including QT prolongation and reduced LVEF, were mitigated by monitoring, but dose-limiting cardiotoxicity remained a constraint. Rare immune-related adverse events occurred when combined with checkpoint inhibitors [300,301]. Pharmacokinetic limitations also posed challenges: a short half-life of 4–6 h required twice-daily dosing, while plasma exposure above ~300 mg/day was associated with increased cardiac toxicity [262,302].

Despite promising mechanisms, clinical efficacy was modest. In a phase II HCC trial, galunisertib monotherapy achieved only a 5% response rate and median PFS of 2.7 months, failing to improve overall survival compared to sorafenib [303]. In pancreatic cancer, its combination with gemcitabine yielded an 11% response rate and median PFS of 3.6 months [304]. Most studies lacked validated biomarker-driven stratification, limiting the identification of responsive subgroups.

The clinical development of galunisertib underscores the complexity of TGF-β as a dual tumor suppressor and promoter. While its pharmacological inhibition showed proof of concept for targeting EMT, angiogenesis, and fibrosis in cancer, challenges with pharmacokinetics, safety, and lack of predictive biomarkers restricted its impact. Lessons from galunisertib inform the design of next-generation TGF-βRI inhibitors, highlighting the need for optimized drug properties, biomarker-guided patient selection, and rational combination regimens to realize the therapeutic potential of TGF-β pathway blockade.

**Figure 7:**
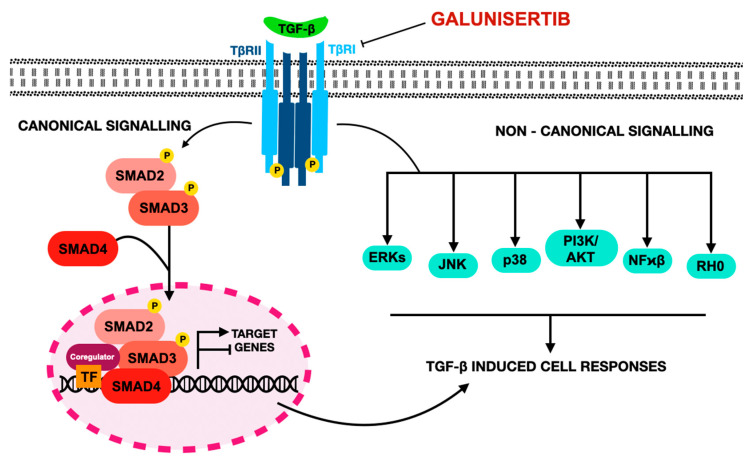
Mechanism of action of galunisertib according to [305]. TGF-β is a protein that plays a crucial role in regulating cell growth, differentiation, and immune responses. In cancer, abnormal TGF-β signaling can promote tumor growth, invasion, and immune evasion. Targeting TGF-β signaling in cancer treatment involves blocking the pathway that transmits signals from the TGF-β protein to the inside of the cell. This can be done by inhibiting downstream signaling components, such as the kinase activity of the TGF-β receptor. Small molecule kinase inhibitors like galunisertib work by blocking this receptor’s activity (targeting TβRI kinase), preventing the cancer-promoting signals from being carried out. Mature TGF-β binds to type I and type II serine/threonine kinase receptors (TβRI and TβRII), forming a heterotetrameric complex. The constitutively active TβRII phosphorylates and activates TβRI, which then initiates the canonical TGF-β signaling pathway. Activated TβRI phosphorylates SMAD family of proteins: SMAD2 and SMAD3 at their C-terminal serine residues. These phosphorylated SMADs form a trimeric complex with SMAD4, translocate to the nucleus, and regulate gene expression in cooperation with other transcription factors. Beyond transcriptional regulation, SMADs influence gene expression through epigenetic changes, RNA splicing, and miRNA processing. All abbreviations employed are defined in the text in the Abbreviations section.

Figure 7 illustrates the mechanism of action of galunisertib.

## 5. Future Perspectives and Clinical Implications in EMP Modulation

Key limitations of galunisertib—namely its short half-life necessitating intermittent dosing, the occurrence of cardiac toxicities, and the absence of validated predictive biomarkers—have guided the development of more advanced therapeutic strategies [248,249,250,251]. One such innovation is the design of bifunctional molecules like bintrafusp alfa (M7824), which simultaneously blocks PD-L1 and sequesters TGF-β ligands. This dual mechanism represents a refined therapeutic approach, with early-phase trials in NSCLC and biliary tract cancers reporting ORRs of 15–20% and median PFS of 2–4 months [306]. Compared with first-generation inhibitors, bintrafusp alfa has demonstrated improved pharmacokinetics and distinctive immunomodulatory effects.

Parallel efforts are focused on next-generation TGF-βRI inhibitors with greater selectivity and tolerability. Vactosertib (TEW-7197), an orally bioavailable ALK5 inhibitor, counteracts TGF-β–driven EMT and immune suppression and is currently under evaluation in combination with chemotherapy and immune checkpoint inhibitors [307,308]. Clinical programs exploring vactosertib-based regimens aim to enhance pharmacodynamic efficacy while minimizing off-target toxicities [309]. Similarly, LY3200882, another next-generation small-molecule inhibitor, offers improved pharmacokinetic and safety profiles and is in clinical testing for glioblastoma and other solid tumors [310,311]. Together, bifunctional TGF-β traps and selective TGF-βRI inhibitors exemplify a strategic evolution in pathway modulation, informed directly by clinical and pharmacologic lessons from galunisertib.

The MET, AXL, and TGF-β signaling pathways converge into a context-dependent network governing EMP, tumor progression, and therapeutic resistance. In vitro studies show that TGF-β upregulates AXL to stabilize mesenchymal states, while MET activation facilitates colonization during metastasis [125,148]. This has established a canonical model where TGF-β and AXL drive EMT and invasion, whereas MET promotes epithelial re-differentiation at metastatic sites.

However, in vivo and clinical evidence suggests this model is overly simplistic. Under hypoxic conditions or in stiff extracellular matrices, MET activation has been shown to sustain mesenchymal transcriptional programs, motility, and invasiveness, rather than inducing epithelial reversion [101,122]. These findings highlight that EMT/MET dynamics are highly context dependent, shaped not only by intrinsic signaling but also by biomechanical and metabolic cues within the TME. Untangling these context-specific influences is essential for translating preclinical data into clinical impact.

MET, AXL, and TGF-β converge on PI3K/AKT, MAPK, and STAT pathways, forming feedback loops that reinforce tumor plasticity and resistance, while hypoxia, inflammatory cytokines, and stromal inputs further amplify partial EMT states and immunosuppressive niches [247,257]. Yet, much of this understanding arises from 2D culture systems that treat EMT as binary, overlooking hybrid phenotypes and spatial heterogeneity uncovered by single-cell and spatial transcriptomics [245,256]. These limitations restrict translational fidelity and underscore the need for models that capture tumor architecture and microenvironmental complexity.

Beyond plasticity, this signaling triad directly fosters immune evasion. TGF-β excludes cytotoxic T-cell infiltration and expands regulatory T cells, while AXL upregulates PD-L1 and suppresses antigen presentation [42,286]. Mesenchymal-like tumor cells further secrete immunosuppressive cytokines, collectively establishing an immune-evasive TME that compromises immunotherapy efficacy.

Therapeutic strategies are therefore moving toward dual targeting of EMT/MET pathways and immunosuppressive signaling. Ongoing trials are investigating combinations of galunisertib (TGF-β inhibition), bemcentinib (AXL inhibition), and capmatinib (MET inhibition) with immune checkpoint blockade, aiming to overcome resistance and improve patient outcomes.

Looking ahead, integration of EMT-targeting agents with immunotherapy is expected to become more systematic. Agents such as capmatinib, bemcentinib, and galunisertib hold potential to relieve immunosuppressive pressures and sensitize tumors to checkpoint blockade [42,286,306]. Liquid biopsy biomarkers—including circulating tumor DNA (ctDNA), circulating tumor cells (CTCs), and EMT-associated extracellular vesicles—offer minimally invasive means of tracking EMT dynamics, guiding patient selection, and monitoring treatment responses [117,219,256,312]. Emerging model systems, such as organoids and single-cell platforms, are becoming indispensable tools to interrogate tumor heterogeneity, hybrid states, and context-specific drug effects [53,245,269,313].

Patient populations most likely to benefit from EMT-targeting approaches include those with fibrotic, TGF-β–rich tumors (e.g., HCC, PDAC, NSCLC), patients resistant to immunotherapy due to T-cell exclusion, and tumors with high EMT-TF expression signatures detected via liquid biopsy or transcriptomic profiling [256,269,306]. Capmatinib is particularly suited to MET-driven subsets such as NSCLC with MET exon 14 skipping mutations or MET amplification [38,41,44]. Bemcentinib may benefit AXL-overexpressing tumors associated with resistance and immune evasion [50,142,145]. Galunisertib and newer TGF-βRI inhibitors are promising for fibrotic and TGF-β–driven cancers, where they can remodel stroma, reverse EMT, and restore immune infiltration, especially in combination with chemotherapy or immunotherapy [239,243,269].

Altogether, this integrated paradigm emphasizes precision oncology: combining EMT-targeting agents with immune modulation, guided by dynamic biomarker assessments and advanced preclinical models, to optimize patient-specific efficacy and overcome adaptive resistance [247,257,306].

Table 5 summarizes representative targeted small-molecule inhibitors of EMT-related pathways in cancer therapy, including capmatinib, bemcentinb, and galunisertib.

## 6. Conclusions

MET is a fundamental biological process with broad implications for targeted cancer therapy. The reciprocal interplay between EMT and MET underlies cellular plasticity, driving both metastatic progression and therapeutic resistance. Whereas EMT enables local invasion and dissemination, MET is essential for metastatic colonization and expansion at distant sites. This bidirectional adaptability highlights the therapeutic potential of MET modulation within a comprehensive oncologic framework.

Agents such as galunisertib, capmatinib, and bemcentinib illustrate complementary strategies for targeting MET-related pathways. Galunisertib inhibits TGF-βRI, attenuating a key EMT-inducing signal and indirectly favoring MET. Capmatinib blocks c-Met receptor tyrosine kinase, disrupting survival and EMT-associated signaling. Bemcentinib targets AXL, reducing mesenchymal traits and overcoming resistance. Despite distinct mechanisms, these agents converge on the principle that restraining EMP can limit plasticity and sensitize tumors to treatment.

Nonetheless, major challenges remain. Most clinical trials lack biomarker-driven designs to identify patients most likely to benefit from EMP-targeting agents. Longitudinal tracking of EMP dynamics in patients is limited, constraining insights into how plasticity evolves under therapeutic pressure. Resistance mechanisms, including pathway crosstalk and adaptive reprogramming, continue to compromise the durability of these strategies.

Future progress will require addressing these gaps. Incorporating EMP modulation into trial endpoints may offer more precise measures of therapeutic benefit beyond survival outcomes. Rational combinations—such as EMP-targeting agents with immunotherapy, chemotherapy, or anti-angiogenic drugs—hold promise for overcoming compensatory resistance. The development of predictive biomarkers, including EMP-specific molecular signatures, will be critical for patient stratification and treatment personalization.

Emerging technologies such as single-cell sequencing, real-time molecular profiling, and patient-derived models will deepen understanding of EMP heterogeneity and temporal dynamics. Novel delivery approaches, including nanoparticle-based systems, may enhance the specificity and tolerability of MET inhibitors.

Overall, MET represents a vital yet underutilized therapeutic axis in oncology. By closing current knowledge gaps and strategically integrating EMP-targeted interventions into precision medicine, MET-directed therapies have the potential to play a central role in improving outcomes and survival.

## Figures and Tables

**Table 1:** The role of epithelial–mesenchymal transition transcription factors (EMT-TF) in tumour cell motility (TCM) according to [70]. All abbreviations employed are defined in the text in the Abbreviations section.

EMT-TF	Family	Key Functions	Regulation	Target Genes & Pathways	Impact on Cancer Biology
SNAIL1/2	Zinc finger (SNAIL family)	Repress epithelial genes (e.g., E-cadherin); Suppress tight junction proteins (claudins, occludins, ZO-1, connexins); Inhibit CRUMBS3 (affects polarity)	Activated by TGF-β, EGF, IGF-1, HGF, Wnt/β-catenin, NOTCH; Regulated by GSK3 (degradation); PAK1 phosphorylation (promotes nuclear localization)	E-cadherin, MMPs (context-dependent); CRUMBS3; Alters glucose metabolism (glycolysis shift)	Promotes stemness, invasion, therapy resistance; Associated with poor prognosis and recurrence
TWIST1/2	bHLH	Downregulates E-cadherin; Upregulates fibronectin, N-cadherin, vimentin; Supports stemness and invasion	Regulated by TGF-β2, AKT2, PDGFR; Enhanced by MAPK/AKT phosphorylation	E-cadherin, fibronectin, N-cadherin, vimentin; Controls TGF-β2, AKT2, PDGFR;	Drives EMT and metastasis; Enhances cancer motility and stem-like traits
ZEB1/2	Zinc finger (E-box-binding homeobox)	Represses epithelial genes (e.g., E-cadherin); Activates mesenchymal genes; Represses polarity genes (CDH1, Lgl2, PATJ, Crumbs3)	Induced by estrogen, TGF-β, Wnt/β-catenin-Modulated by SNAIL1 and TWIST1	E-cadherin, MMPs, polarity genes	Promotes EMT and metastasis; Associated with therapy resistance and poor prognosis

**Table 2:** Selected cytoplasmic MET (cMET) inhibitor compounds and their mechanisms of action on the cMET pathway along with corresponding to each type of cMET pathway inhibition, according to [142]. All abbreviations employed are defined in the text in the Abbreviations section.

Mechanism/ Inhibitor	Target/ Pathway Activity	Effect on cMET Signaling	Clinical Relevance/ Notes
Selective cMET TKI; Direct cMET inhibition (*Capmatinib*)	Inhibits cMET, GAB1, SRC, PI3K; blocks AKT, MAPK	Prevents activation of downstream signaling	Approved for NSCLC with MET exon 14 skipping mutations
Selective cMET inhibitor; Direct inhibition (*Savolitinib*)	Inhibits cMET phosphorylation; blocks survival and proliferation pathways	Inhibits cMET downstream signaling	Investigated in NSCLC and gastric cancer
Selective cMET inhibitor; Direct inhibition (*Tepotinib*)	Prevents cMET dimerization and phosphorylation; inhibits STAT3, PI3K/AKT, MAPK	Prevents cMET signaling and downstream cascade activation	Approved for NSCLC with MET exon 14 skipping mutations
Monoclonal antibody; HGF binding inhibitor (*Onartuzumab*)	Blocks HGF binding; prevents receptor dimerization and downstream PI3K, RAS/MAPK signaling	Prevents receptor activation at extracellular level	Explored in clinical trials; limited efficacy alone, combined therapies being explored
Multi-kinase inhibitor (cMET, VEGFR, AXL) (*Cabozantinib*)	Inhibits cMET and angiogenesis pathways (VEGFR)	Inhibits tumor growth via cMET and VEGFR pathways	Approved for renal cell carcinoma and thyroid cancer
Multi-targeted TKI (cMET, VEGFR, RON) (*Foretinib*)	Inhibits migration/invasion pathways (FAK, RAC1/JNK)	Broad inhibition including cMET, VEGFR pathways	Evaluated in trials for gastric and other cancers
Non-ATP competitive cMET inhibitor (*Tivantinib*)	Disrupts cMET signaling; inhibits survival/proliferation; mechanism partially unclear	Blocks downstream effects despite unclear kinase binding	Clinical trials in lung and liver cancers
TKI; Direct cMET kinase inhibition (*Crizotinib*)	Inhibits cMET autophosphorylation; blocks AKT/mTOR, RAS/RAF/MAPK, STAT3	Prevents downstream signaling cascades	Approved for NSCLC with cMET alterations; also inhibits ALK

**Table 3:** Targeting Aberrantly Activated AXL to attenuate EMT and overcome drug resistance according to [223]. All abbreviations employed are defined in the text in the Abbreviations section.

Feature	Targeting Aberrantly Activated AXL
Key Inhibitors	Bemcentinib (AXL inhibitor), Selumetinib (MEK inhibitor combo)
Primary Cell Type Targeted	Mesenchymal cells (AXL), epithelial cells (MEK inhibitor)
Mechanism of Action	Blocks AXL-driven EMT signaling activated by TGF-β and hypoxia
Effect on EMT	Attenuates TGF-β and hypoxia-induced EMT
Impact on Drug Resistance	Restores TKI sensitivity, reduces growth of EMT and drug-resistant tumors
Combination Strategies	Combined with MEK inhibitor selumetinib for dual epithelial and mesenchymal targeting
Apoptosis Induction	Indirect via EMT attenuation
Clinical Implications	Promising for overcoming resistance in tumors with EMT and drug resistance
Challenges	Requires targeting multiple pathways due to EMT complexity

**Table 4:** TGFβ pathway overview according to [247]. All abbreviations employed are defined in the text in the Abbreviations section.

Stage/Component	Details
Ligand Interaction	The function of TGFβ ligands is influenced by the variety and concentration of ligands and receptors present, alongside extracellular inhibitors and accessory molecules. Proteins like Gremlin 1 (GREM1) can suppress related signaling pathways (such as BMP), affecting cellular characteristics. Co-receptors like β-glycan and CRIPTO facilitate or hinder receptor activation based on context.
Receptor Complex Formation	TGFβ receptors assemble as heterotetramers comprising type I and type II subunits, each with multiple variants. Binding of ligands prompts type II receptors to phosphorylate type I receptors, initiating downstream signaling. The specific pairing of receptor subtypes with ligands dictates the cellular response. Regulatory factors like FKBP12 and BAMBI inhibit receptor activation to fine-tune signaling output.
Canonical Intracellular Signaling	Activated type I receptors phosphorylate receptor-specific SMAD proteins (R-SMADs), which combine with SMAD4 to form complexes that translocate to the nucleus. Different R-SMADs respond to TGFβ-like or BMP-like signals, while inhibitory SMADs (SMAD6/7) suppress the pathway. Scaffold proteins such as SARA regulate SMAD phosphorylation and activation.
Non-Canonical Intracellular Pathways	In addition to SMAD-dependent signaling, TGFβ receptors engage alternative pathways including MAPK cascades (JNK, p38, ERK), PI3K-AKT signaling, and Rho GTPase activation. These non-canonical routes interact with SMAD signaling to shape cellular outcomes, though their roles depend on the specific physiological or pathological setting.
Nuclear Regulation	SMAD complexes are imported into the nucleus via specialized transport proteins, where they partner with cell-specific transcription factors to control gene expression. They also recruit chromatin remodeling enzymes that either promote or repress transcription. Alternative SMAD interactions, such as with TRIM33, facilitate opening of chromatin regions critical for differentiation and development.

**Table 5:** Selected targeted small-molecule inhibitors of EMT pathways in cancer therapy on the examples of comparison of capmatinib, bemcentinib and galunisertib according to [314,315,316,317,318,319,320,321]. All abbreviations employed are defined in the text in the Abbreviations section.

Feature/Drug	Capmatinib (Tabrecta^®^)	Bemcentinib (BGB324)	Galunisertib (LY2157299)
Mechanism of Action	Selective MET tyrosine kinase inhibitor; targets MET exon 14 skipping mutations	Selective AXL receptor tyrosine kinase inhibitor; blocks EMT, metastasis, immune evasion	Oral inhibitor of TGF-β receptor I kinase (ALK5); suppresses TGF-β-driven EMT and immune evasion
Clinical Applications	Approved for metastatic NSCLC with MET exon 14 skipping mutations	NSCLC (especially STK11-mutant), AML, MM	CRC/RC, HCC, GBM, MDS, NSCLC, PC
Clinical Trials	GBM (NCT02386826), NSCLC (NCT04427072, NCT01911507, NCT05435846)	AML (NCT02488408, NCT03824080), MM (MiST3 NCT03654833), NSCLC (NCT05469178, NCT03184571, NCT02424617)	ASolT (NCT01682187), CRC (NCT05700656), HCC (NCT02240433), GBM (NCT01582269), PC (NCT02734160), PCa (NCT02452008), RC (NCT02688712)
Common Adverse Effects	Peripheral edema, nausea, fatigue, vomiting, dyspnea, elevated liver enzymes, pancreatitis, interstitial lung disease (ILD)	Elevated liver enzymes, fatigue, diarrhea, anemia, thrombocytopenia, QTc prolongation	Fatigue, diarrhea, nausea, vomiting, constipation, transaminase elevation, cytopenias, thrombosis, neutropenia, dyspnea, hypophosphatemia, hand-foot syndrome
Regulatory Status	FDA-approved for METex14 NSCLC	Fast Track designation by FDA for elderly relapsed AML	Investigational; in various phase trials for multiple cancers
Efficacy	High in METex14-mutated NSCLC; ORR ~68% in treatment-naïve patients	Modest; ORR ~46% in NSCLC with pembrolizumab	Variable; improved OS in pancreatic cancer with gemcitabine
Toxicity	Generally mild; nausea, fatigue, peripheral edema	Mild to moderate; rash, diarrhea, fatigue, elevated liver enzymes	Mild; no dose-limiting toxicities reported
Resistance	Low; rare mutations reported	Moderate; common in ~20% of cases	High; frequent relapse observed
Key Trial Outcomes	Improved OS and PFS in METex14-mutated NSCLC	No significant improvement in efficacy with combination therapies	Prolonged OS with gemcitabine in pancreatic cancer; minimal added toxicity

## Data Availability

Data sharing is not applicable to this article.

## Abbreviations

The following abbreviations are used in this manuscript:

AKT	Protein kinase B
ALK	Anaplastic lymphoma kinase
ALK5	Activin receptor-like kinase 5
ALT	Alanine aminotransferase
AML	Acute myeloid leukemia
AST	Aspartate aminotransferase
ASolT	Advanced Solid Tumors
ATP	Adenosine triphosphate
AXL	AXL receptor tyrosine kinase
bHLH	Basic helix–loop–helix
CDH1	Gene encoding E-cadherin
CDK(s)	Cyclin-dependent kinase(s)
CIP1	CDK-interacting protein 1 (p21^CIP1^)
CKD	Chronic kidney disease
CNS	Central nervous system
CRC	Colorectal cancer
CRC/RC	Colorectal and rectal cancers
ctDNA	Circulating tumor DNA
CTCs	Circulating tumor cells
CYP3A4	Cytochrome P450 3A4
DNA	Deoxyribonucleic acid
DNMT	DNA methyltransferase
ECM	Extracellular matrix
EGF(R)	Epidermal growth factor (receptor)
EMP	Epithelial–mesenchymal plasticity
EMT	Epithelial–mesenchymal transition
EMT TFs	Epithelial–mesenchymal transition transcription factors
ERK/ERKs	Extracellular signal-regulated kinase(s)
FAK	Focal adhesion kinase
FGFR1	Fibroblast growth factor receptor 1
GAB1	GRB2-associated binder 1
GAS6	Growth arrest–specific 6
GBM	Glioblastoma
GRB2	Growth factor receptor–bound protein 2
GSK3	Glycogen synthase kinase 3
GTPase	Guanosine triphosphatase
HCC	Hepatocellular carcinoma
HDAC	Histone deacetylase
HGF	Hepatocyte growth factor
HER3	Human epidermal growth factor receptor 3
IHC	Immunohistochemistry
ILD	Interstitial lung disease
INK4b	Inhibitor of CDK4 (p15^INK4b^)
JNK	c-Jun N-terminal kinase
Lgl2	Lethal giant larvae homolog 2
LVEF	Left ventricular ejection fraction
MAPK	Mitogen-activated protein kinase
MDS	Myelodysplastic syndromes
MEK	Mitogen-activated protein kinase kinase
MET	Mesenchymal–epithelial transition/MET receptor tyrosine kinase
miR/miRNA	MicroRNA
MM	Malignant Mesothelioma
MMP/MMPs	Matrix metalloproteinase(s)
MPNs	Myeloproliferative neoplasms
MRONJ	Medication-related osteonecrosis of the jaw
mRNA	Messenger RNA
mTOR	Mechanistic target of rapamycin
NF-κB	Nuclear factor kappa-light-chain-enhancer of activated B cells
NSCLC	Non-small cell lung cancer
ORR	Objective response rate
PAK1	p21-activated kinase 1
PATJ	PALS1-associated tight junction protein
PC	Pancreatic cancer
PCa	Prostate cancer
PDAC	Pancreatic ductal adenocarcinoma
PDGFR	Platelet-derived growth factor receptor
PFS	Progression-free survival
PI3K	Phosphatidylinositol 3-kinase
PLCγ	Phospholipase C gamma
PTEN	Phosphatase and tensin homolog
QTc	the corrected QT interval
RAC1	Ras-related C3 botulinum toxin substrate 1
RAF	Rapidly accelerated fibrosarcoma kinase
RAS	Rat sarcoma virus oncogene
RC	Rectal cancer
RHO	Ras homologous GTPase
RNA	Ribonucleic acid
RON	Recepteur d’origine nantais (RON receptor tyrosine kinase)
RTK(s)	Receptor tyrosine kinase(s)
SHC	SHC-transforming protein
SHP2	Src homology region 2-containing protein tyrosine phosphatase 2 (PTPN11)
SLUG	Snail family transcriptional repressor 2
SNAIL/SNAIL1/2	Zinc finger transcription factors repressing E-cadherin
SMAD	Transcriptional mediators of TGF-β receptor signaling
SOS	Son of Sevenless (guanine nucleotide exchange factor)
SRC	Proto-oncogene tyrosine-protein kinase Src
STAT/STAT3	Signal transducer and activator of transcription
STK11	Serine/threonine kinase 11
TAM	TYRO3, AXL, MER receptor tyrosine kinase family
TGF-β(R)/(RI)	Transforming growth factor-beta (receptor/receptor I)
TKI	Tyrosine kinase inhibitor
TNF-α	Tumor necrosis factor-alpha
TP53	Tumor protein p53
Tregs	Regulatory T cells
TWIST/TWIST1/2	EMT-inducing transcription factors
VEGF(R)	Vascular endothelial growth factor (receptor)
ZEB1/2	Zinc finger E-box-binding homeobox 1/2
ZO-1	Zonula occludens-1
